# Is SARS-CoV-2 Vertically Transmitted?

**DOI:** 10.3389/fped.2020.00276

**Published:** 2020-05-15

**Authors:** Ana Cristina Simões e Silva, Caio Ribeiro Vieira Leal

**Affiliations:** ^1^Interdisciplinary Laboratory of Medical Investigation, Faculty of Medicine, Federal University of Minas Gerais (UFMG), Belo Horizonte, Brazil; ^2^Obstetrics/Gynecology Department, Clinics Hospital, Federal University of Minas Gerais (UFMG), Belo Horizonte, Brazil

**Keywords:** SARS-CoV-2, COVID-19, vertical transmission, pregnant women, neonate, intrauterine infection

## Abstract

At the end of 2019, in Wuhan (China), the onset of a disease caused by severe acute respiratory syndrome coronavirus 2 (SARS-CoV-2) was observed. The disease, named COVID-19, has a wide spectrum of clinical presentations, ranging from asymptomatic or mild to critical, and for some patients the disease is even fatal. Apparently, being a child or being pregnant does not represent an additional risk for adverse outcomes. The purpose of this mini-review was to investigate what is in the scientific literature, so far, in regard to vertical transmission of SARS-CoV-2. Data were obtained independently by the two authors, who carried out a systematic search in the PubMed, Embase, LILACS, Cochrane, Scopus and SciELO databases using the Medical Subject Heading terms “coronavirus,” “COVID-19,” and “vertical transmission.” Few studies about the vertical transmission of SARS-CoV-2 are found in the literature. In all case reports and case series, the mothers' infection occurred in the third trimester of pregnancy, there were no maternal deaths, and most neonates had a favorable clinical course. The virus was not detected in the neonate nasopharyngeal swab samples at birth, in the placenta, in the umbilical cord, in the amniotic fluid, in the breast milk or in the maternal vaginal swab samples in any of these articles. Only three papers reported neonatal SARS-CoV-2 infection, but there is a bias that positive pharyngeal swab samples were collected at 36 h and on the 2nd, 4th, and 17th days of life. The possibility of intrauterine infection has been based mainly on the detection of IgM and IL-6 in the neonates' serum. In conclusion, to date, no convincing evidence has been found for vertical transmission of SARS-CoV-2.

## Introduction

At the end of 2019, in Wuhan (China), the onset of a disease caused by *severe acute respiratory syndrome coronavirus 2* (SARS-CoV-2) was observed. SARS-CoV-2 caught the attention of the entire world due to its great potential for dissemination in a short time and soon gained the status of a public emergency of international concern. As of March 31, 2020, the World Health Organization (WHO) has reported a total of 750,890 cases and 36,405 deaths related to SARS-CoV-2 infection on its official website^1^.

The disease associated with SARS-Co-V-2 infection, designated by the WHO as COVID-19, has a wide spectrum of clinical presentations, ranging from asymptomatic or mild to critical, and for some patients the disease is even fatal. Most fatal cases have occurred in individuals with advanced age or with underlying medical conditions, including cardiovascular diseases, diabetes, and hypertension, among others (1). Apparently, being a child or being pregnant does not represent an additional risk for adverse outcomes (2).

SARS-CoV-2 is part of the family *Coronaviridae*, a family of enveloped, positive single-stranded large RNA viruses, which also includes *severe acute respiratory syndrome coronavirus* (SARS-CoV), discovered in 2003 (3), and *Middle East respiratory syndrome coronavirus* (MERS-CoV), discovered in 2012 (4). The viruses have bats and other mammals as natural reservoirs. Animal-human and human-human transmissions are very fast. Both viruses came into evidence after two major outbreaks of respiratory diseases, in China, in 2002–2003 for SARS-CoV and, in the Middle East, in 2012, for MERS-CoV. The mortality rates were estimated to be over 10% for SARS-CoV infection and >35% for MERS-CoV infection (5). Most coronaviruses are viruses that are highly pathogenic and have the potential to produce serious infections of the lower respiratory tract. Unlike what is observed among those infected with SARS-CoV-2, pregnant patients infected with SARS-CoV tend to have a high rate of adverse outcomes when compared to no pregnant women (6). However, no proven cases of vertical transmission of SARS-CoV or MERS-CoV have yet been described (7, 8). In this context, the purpose of this text was to investigate what is in the scientific literature, so far, in regard to the possibility of vertical transmission of SARS-CoV-2.

## Methods

Data were obtained independently by the two authors, who carried out a comprehensive and systematic search in the PubMed, Embase, LILACS, Cochrane, Scopus and SciELO databases. Search strategies included the Medical Subject Heading terms “coronavirus,” “COVID-19,” and “vertical transmission.” The filters used were the reading of the title and abstract of the articles. The articles obtained were case reports or case series of women infected with SARS-CoV-2 during pregnancy or of neonates born to infected mothers. We found 10 articles to be included for a critical analysis in this review (9–18).

## Results

Due to the recent nature of the disease, few studies are found in the literature about the vertical transmission of SARS-CoV-2. In all case reports and case series, the mothers' infection occurred in the third trimester of pregnancy, there were no maternal deaths, and most neonates had a favorable clinical course. The methodology varied among studies, but in most articles, serum samples and swabs from the newborn's pharynx, samples of breast milk and samples of products of conception (placenta, amniotic fluid and umbilical cord blood) were collected for further laboratory testing (9–18). The main characteristics of each study are shown in Table 1. With the exception of two patients (17), all had cesarean section deliveries and without skin-to-skin contact with the newborn in the delivery room. Only in the study by Zhu et al. (17) was there a neonatal death. The case was a male newborn with a gestational age of 34 + 5/7 weeks. The newborn stayed in the hospital from the first day of life due to respiratory distress, and his condition deteriorated on the eighth day of life to refractory shock, multiple organ failure and disseminated intravascular coagulation; he died on the ninth day of life. The nasopharyngeal swab of this newborn, collected at birth, was negative for SARS-CoV-2. In all other studies, there were no fetal deaths, neonatal deaths or cases of severe intrauterine asphyxia. The virus was not detected in the neonate nasopharyngeal swab samples at birth, in the placenta, in the umbilical cord, in the amniotic fluid, in the breast milk or in the maternal vaginal swab samples in any of these articles (9–18). Only one study showed a SARS-CoV-2-positive pharynx swab, but the sample was collected at 36 h of age (13). Additional results were also found and were used to support the possibility of vertical infection by SARS-CoV-2 in two studies (14, 15): high levels of IgM for SARS-CoV-2 in the blood of neonates and increased concentrations of cytokines, including IL-6 and IL-10. It is important to mention that products of conception were not tested in these two studies.

**Table 1:** Summary of studies about vertical transmission of SARS-CoV-2.

**References**	**Type of study**	**Main results**
Fan et al. (9)	Case series of two pregnant women infected with SARS-CoV-2 in the third trimester	The virus was not detected in the neonate nasopharyngeal swab samples at birth, in the placenta, in the umbilical cord, in the amniotic fluid, in the breast milk, or in the maternal vaginal swab.
Chen et al. (10)	Case series of 9 pregnant women infected with SARS-CoV-2 in the third trimester	The virus was not detected in the neonate nasopharyngeal swab samples at birth, in the umbilical cord, in the amniotic fluid, or in the breast milk.
Chen et al. (11)	Case series of 3 pregnant women infected with SARS-CoV-2 in the third trimester	The virus was not detected in the neonate nasopharyngeal swab samples at birth or in the placenta.
Liu et al. (12)	Case series of 3 pregnant women infected with SARS-CoV-2 in the third trimester	The virus was not detected in the neonate nasopharyngeal swab samples at birth, in the placenta, in the umbilical cord, in the amniotic fluid, in the breast milk, or in the maternal vaginal swab.
Wang et al. (13)	Case report of a neonate infected with SARS-CoV-2	The pharynx swab at 36 h of age waspositive for SARS-CoV-2. It was not possible to collect a pharynx swab at birth. The virus was not detected in the placenta, breast milk or umbilical cord.
Zeng et al. (14)	Case series of 6 pregnant women infected with SARS-CoV-2 in the third trimester	The virus was not detected in neonates' nasopharyngeal swab or serum samples at birth. However, 2 newborns had elevated levelsof IgM for SARS-CoV-2, and there were elevated levelsof IL-6 in all 6 newborns. No other product of conception was tested.
Dong et al. (15)	Case report of a neonate infected with SARS-CoV-2	The virus was not detected in the neonate nasopharyngeal swab samples at birth. There were elevated levelsof IgM, IL-6 and IL-10 in the serum sample at 2 h of age. No other product of conception was tested.
Li et al. (16)	Case report of 1 pregnant woman infected with SARS-CoV-2 in the third trimester	The virus was not detected in the neonate nasopharyngeal swab samples at birth, in the umbilical cord, in the amniotic fluid, or in the breast milk.
Zhu et al. (17)	Case report of 10 neonates born to mothers infected with SARS-CoV-2in the third trimester	The virus was not detected in the neonates' nasopharyngeal swabs1 to 9 days after birth.
Chen et al. (18)	Case report of 4 neonates born to mothers with COVID-19	Three neonates had negative results in nasopharyngeal swab tests for the virus. In one neonate, the nasopharyngeal swab test was not performed. None of them developed serious clinical symptoms and all were well at the time of hospital discharge.
Breslin et al. (19)	Case report of 18 neonates born to mothers infected with SARS-CoV-2	The virus was not detected in the neonate nasopharyngeal swab samples at birth or at 1 or 2 days of life.
Zeng et al. (20)	Case report of 33 neonates born to mothers infected with SARS-CoV-2	The nasopharyngeal swab samples taken at 2 and 4 days of life werepositive for SARS-CoV-2 in only 3 neonates (9%).

Concerning the outcomes of pregnant women and their newborns, these studies did not report deaths of the mothers, and most of the newborns were discharged in good health conditions (9–20).

## Discussion

After analyzing these studies, no convincing evidence was found for vertical transmission of SARS-CoV-2 in pregnant women infected during the third trimester of pregnancy, as also reported for SARS-CoV infection (21). SARS-CoV-2 was not detected in any of the patients analyzed in these papers in the amniotic fluid, placenta or umbilical cord using the reverse-transcription polymerase chain reaction (RT-PCR) technique. Currently, the RT-PCR technique in a sample of respiratory tract secretions—nasopharyngealswabs, sputum or bronchoalveolar lavage, for example—is considered the gold standard for the diagnosis of SARS-CoV-2 infection due its high specificity (22). However, the method presents some limitations, including the non-negligible number of false-negative results, the time necessary to obtain results and the need forspecialized equipment to perform the test (23). A positive RT-PCR test usually confirms the diagnosis, but in the case of a negative test when infection is very probable, samples from other sites in the respiratory tract should be analyzed, according to WHO guidelines, to increase the accuracy (24). False-negative tests can occur due to the limit of detection (LoD), which is the lowest concentration of viral RNA that can be detected by the technique at least 95% of the time (25).

There was no report of positivity of the nasopharyngeal swab PCR test of neonates at birth. Only three papers (13, 20, 26) reported neonatal SARS-CoV-2 infection, but there is a bias that positive pharyngeal swab samples were collected at 36 h and on the 2nd, 4th, and 17th day of life. Therefore, the possibility of nosocomial infection cannot be ruled out. In general, infants born to mothers with COVID-19 have a favorable clinical course (9–20).

An interesting issue to be analyzed is the difference in the clinical course between pregnant patients infected with SARS-CoV-2 and those infected with SARS-CoV. After the SARS-CoV epidemic that occurred in 2003–2004 in Asia, some studies showed that the infection led to some unfavorable outcomes in pregnant women, such as preterm delivery, spontaneous abortions and restricted intrauterine growth (27). The most recent data show that fetal complications related to SARS-CoV-2 maternal infection exist, but the rates are not high, with an estimated rate of miscarriage of ~2% and of restricted intrauterine growth of ~10% (28). In the case of SARS-CoV infection (27), a review (7) regarding the possibility of vertical transmission ofSARS-CoV was carried out based on a case series (29) including a total of 12 pregnant women: 7 infected during the first trimester and 5 infected during the second or third trimesters. In the first group, 4 women had spontaneous abortions. In the second group, all had live births, but 3 needed urgent cesarean sections; in those two who did not require early obstetric intervention, oligohydramnios and severe fetal growth restriction were found. The virus was not found in samples of amniotic fluid, blood culture of the newborn or endotracheal aspirate of the newborn in any of the patients. None of the neonates showed dysmorphisms at birth. In addition, all of the neonates exhibited a clinical course similar to that of other neonates under the same clinical conditions (7).

A recent editorial by Kimberlin and Stagno (30) discussed two articles (14, 15) that raised the possibility of intrauterine infection by showing high levels of IgM for SARS-CoV-2 and the cytokine IL-6 in neonate serum. In the case series of Zheng et al. (14), none of the 6 neonates had a SARS-CoV-2-positive nasopharynx swab at birth, nor was the virus identified in the serum, but 2 samples showed SARS-CoV-2-positive IgM, and in all samples, there were high levels of IL-6. In the case report by Dong et al. (15), there were high levels of IgM for SARS-CoV-2 and IL-6 and IL-10 at 2 h of life, but the nasopharynx swab was negative. The editorial then questions the reliability of IgM detection to determine intrauterine infection. Due to its molecular mass, IgM generally does not cross the placental barrier in large quantities, but the transfer of some types of immunoglobulins that do not normally cross the placental barrier (such as IgM or IgA) can happen in normal situations, even in small quantities, and this can be intensified in special situations, such as the inflammation of the birth canal (31). In addition, tests for the detection of IgM frequently present false-negative and false-positive results. For example, the first-generation IgM enzyme-linked immunosorbent assay test had a sensitivity of ~70% and a specificity of ~95% for congenital cytomegalovirus infection (30). The editorial also points out that the sharp decline in IgM levels in a short time does not show the same behavior as that shown for other congenital infections, such as rubella or Zikavirus infection (29).

It should also be noted that the cytokine IL-6 is a soluble mediator of the immune system response. IL-6 stimulates the body's defense response in several situations, including infections or autoimmune diseases. Its action on the pathogenesis of COVID-19 has been studied recently, and its measurement may have a fatality prediction value in adult patients (32). However, as with IgM, the assessment of IL-6 levels in neonates cannot be considered a good standard for the determination of whether vertical transmission of SARS-CoV-2 occurs, since IL-6 can pass through the placenta (33).

Thus, we considered that the assumption of vertical transmission of SARS-CoV-2 is not possible based only on the positivity of IgM antibodies or high levels of IL-6 in the neonate. Further studies are needed to assess the reliability of the assessment of neonatal IgM and other molecules, such as IL-6, in maternal SARS-CoV-2 infection. In addition, in the case series of Zheng et al. (14) and in the case report of Dong et al. (15), the virus was not detected in any laboratory examination, including nasopharynx swab of the newborn at birth, or in any product of conception.

## Conclusions

In summary, unlike pregnant women infected with other coronaviruses (SARS-CoV or MERS-CoV) (7, 8, 17, 34), those infected with SARS-CoV-2 are not prone to unfavorable pregnancy outcomes. Additional studies are needed to assess whether there is in fact vertical transmission of the virus. To date, the possibility of intrauterine infection has been based mainly on the detection of IgM and IL-6 in neonates' serum. Studies that detected the virus in neonatal nasopharyngeal swabs did so hours or days after birth; therefore, the possibility of nosocomial infection cannot be ruled out. In addition, the virus was not detected in products of conception or breast milk. It should also be noted that pregnant women infected with SARS-CoV-2 have the same clinical course as non-pregnant women, and until now, all neonates with suspected COVID-19 due to vertical transmission of SARS-CoV-2 have had, in general, favorable evolution.

## Author Contributions

AS and CL independently performed the search of the literature, analyzed the articles, wrote the manuscript, and approved the final version. AS submitted the manuscript.

## Conflict of Interest

The authors declare that the research was conducted in the absence of any commercial or financial relationships that could be construed as a potential conflict of interest.

## Footnotes

^1^Available online at: https://www.who.int/emergencies/diseases/novelcoronavirus-2019/situation-reports/ (accessed March 31, 2020).
